# Playground Design: Contribution to Duration of Stay and Implications for Physical Activity

**DOI:** 10.3390/ijerph20054661

**Published:** 2023-03-06

**Authors:** Deborah A. Cohen, Meghan Talarowski, Bing Han, Stephanie Williamson, Emily Galfond, Deborah R. Young, Sarah Eng, Thomas L. McKenzie

**Affiliations:** 1 Kaiser Permanente Southern California Research and Evaluation, Pasadena, CA 91101, USA; 2 Studio Ludo, Philadelphia, PA 19143, USA; 3 Research Programming Group, Information Services, RAND Corporation, Santa Monica, CA 90407, USA; 4 School of Exercise and Nutritional Sciences, San Diego State University, San Diego, CA 92182, USA

**Keywords:** playgrounds, physical activity, design

## Abstract

Background: The study goal was to identify playground features associated with visitor length of stay and physical activity. Methods: We observed playground visitors over 4 days during summer 2021 in 60 playgrounds in 10 US cities, selected based on design, population density, and poverty levels. We observed 4278 visitors and documented their length of stay. We observed an additional 3713 visitors for 8 min, recording their playground location, activity level, and use of electronic media. Results: People stayed an average of 32 min (range 5 min–4 h). Stay time varied by group size, with larger groups staying longer. The presence of restrooms increased the likelihood of staying longer by 48%. Playground size, mature trees, swings, climbers, and spinners were associated with longer stays. When a teen was a part of the group observed, the group was 64% less likely to stay longer. The use of electronic media was associated with lower amounts of moderate-to-vigorous physical activity compared to non-media users. Conclusions: To increase population-level physical activity and time spent outdoors, playground features associated with a longer stay should be considered when renovating or building new playgrounds.

## 1. Introduction

While playgrounds are settings primarily designed for children under age 12, they have the potential to help people of all ages to be physically active and engage in diverse movements (e.g., climbing, swinging, jumping, spinning) to meet both aerobic physical activity guidelines and engage in recommended muscle and bone strengthening activities [1]. Because physical activity is higher when people are outdoors than indoors [2,3,4], capitalizing on factors that increase the time spent outdoors may be a promising way to support more physical activity as well as enhance mental health, which has been associated with time spent in nature [5]. Given that physical inactivity is a risk factor for multiple conditions and chronic diseases [6], it is important for society to support infrastructure that helps all local residents achieve the recommended strength and aerobic physical activity guidelines: 60 min per day of moderate-to-vigorous physical activity (MVPA) for children and 150 min per week for adults [7].

Playgrounds with features that appeal to both adults and children could potentially support more physical activity by increasing the frequency of visits and/or duration of time spent there. Some playgrounds are now being built with features that can accommodate people of all ages, so adult caregivers can also be active there [8,9]. As part of the National Study of Playgrounds [10], we observed how visitors used playgrounds, how they engaged with playground features, and if and how they used their personal electronic media (e.g., cell phones/tablets) at the playgrounds. Although being outdoors has benefits, if the time is spent on electronic media, people may not achieve optimal physical activity goals. Physical activity has been declining with the increased use of electronic media [11].

While there is a national standard for MVPA for children and adults as well as recommendations for muscle and bone strengthening exercises, there is considerable controversy about how much time is recommended for people to spend outdoors. One study found that greater than 120 min per week was associated with good health and well-being [12], while another found that 20 min a day outdoors was associated with a reduction in stress biomarkers [13]. Outdoor play promotes curiosity, creativity, critical thinking, and better learning outcomes [14]. Children who spend time in natural settings have less anger and aggression and better impulse control [14]. Studies have demonstrated that outdoor play reduces symptoms of attention-deficit hyperactivity disorder (ADHD) [15], improves eyesight [16], and contributes to better sleep [17]. Playgrounds are placed in outdoor environments and can contribute to time spent outdoors; however, it is not clear what playground features are associated with playground visits. This paper describes playground features that contribute to the length of stay at playgrounds and estimates the proportion of time visitors spent there engaging with electronic media.

## 2. Materials and Methods

In our National Study of Playgrounds, we studied the use of 60 playgrounds in 10 metro areas: Boston, Chicago, Cincinnati, Denver, Houston, Los Angeles/Orange County, Memphis, New York City, San Francisco, and Seattle [10]. Playgrounds were selected to include traditional and innovative designs. 

### 2.1. Selection of Playgrounds

The goal of the parent National Study of Playgrounds was to compare innovatively designed playgrounds to the standard post-and-platform design to understand how design influences use and physical activity. The 30 innovatively designed playgrounds chosen were a convenience sample from 10 localities that had at least three in the area, as these playgrounds are still rare compared to the dominant post-and-platform design. We selected 10 metro areas to represent different parts of the United States: Boston, Chicago, Cincinnati, Denver, Houston, Los Angeles/Orange County, Memphis, New York City, San Francisco, and Seattle. The criterion for innovative playgrounds was having at least 3 of 5 features: (a) a variety of surface types; (b) naturalized and planted areas designed specifically for play; (c) open-ended structures that do not dictate play sequences; (d) loose, movable equipment; and (e) not comprised solely of traditional post-and-platform structures. The 30 standard post-and-platform playgrounds were chosen to match the innovative playgrounds based on neighborhood density and percentage of families in poverty based on the US Census [10]. We mapped all playground features, surfaces, and equipment and defined each unique component as a target area. The total number of target areas per playground ranged from 32 to 151.

We used three protocols to assess playground use. The System for Observing Play and Recreation in Communities (SOPARC) [18,19] is a momentary systematic observation protocol and was used to quantify the number of visitors and the amount of MVPA in each playground. This protocol includes counting the number and characteristics of people in each target area, as well as their level of physical activity 19 different times at different hours over 4 days.

Two other observation protocols that we created, Dwell Time and Play Loop, are described below. Data collectors were hired and trained to conduct each of the three protocols. The training consisted of virtual classroom presentations of the methods, followed by multiple practice sessions in playgrounds. Data collectors were certified as proficient if their observations achieved 80% agreement with another independent observer. Two data collectors were at the playground each day performing different protocols on a prespecified schedule. All observations occurred in the summer of 2021 and only on clement days. Any rainy days were postponed to a similar weekday or the same weekend day with no rain in subsequent weeks.

### 2.2. Dwell Time Protocol

To measure the duration of stay (Dwell Time protocol), we followed visitors entering the playground from the time they arrived until the time they left within a 4-h window for 4 days in each of the 60 playgrounds, half from 10 a.m. to 2 p.m. and the other half from 2 p.m. to 6 p.m. Trained observers initiated their surveillance at playground entryways and followed groups of visitors entering the playground. When a new group entered the playground, observers started a stopwatch and recorded the total number of people in the group and the target area where they first entered the playground. Observers then described up to 3 people in the group in detail, recording their characteristics (e.g., apparent gender, age-group, race/ethnicity, and a distinguishing characteristic, like hat or shirt color), so they could easily be located during future intervals. If the group had more than 3 people with different apparent genders and/or age-groups, observers were asked to choose at least one adult and one child, with the third person being a different gender or age-group than either the adult or child chosen, if possible. Every 10 min, observers located each of the 3 individuals and recorded their target area location. If a group was recorded for the first interval only (i.e., they left before the start of the second time interval), Dwell Time was recorded as 5 min. When the group left during subsequent intervals (e.g., before 20 min or 30 min), their Dwell Time was recorded as 15 and 25 min, respectively.

Between the 10-min Dwell Time intervals, when playgrounds were not crowded, it was sometimes possible to observe up to 3 different groups that entered the playground. We then staggered the observations and assigned each group its own digital stopwatch to guide the timing of observations. When one group left, we replaced it with another. In very busy and large playgrounds, observers may have only followed 1 group at a time, while in others, they were able to observe as many as 18 groups in the 4 h period.

To assess the reliability of the Dwell Time protocol, 2 independent data collectors conducted 15 paired observations. For the reliability testing, the data collectors agreed upon which group members to observe when the group arrived at the playground. Agreement was defined as an exact match between the 2 observers. The sample probability of agreement was calculated and used as the reliability measure. Data collectors were certified if they achieved at least 80% agreement with the paired independent observer. The reliability testing was based on 15 pairs of observations, each of which followed a group consisting of 2 or 3 persons made at 10-min intervals. The agreement percentage at the person-by-time level was very high (95.6% (SE = 1.8%)).

### 2.3. Play Loop Protocol

For the Play Loop protocol, we selected a random sample of adults and children and recorded their apparent demographic characteristics (e.g., age-group, gender, and race/ethnicity), and followed each individual for 8 min. During this protocol, we noted the target areas where the individuals were every 20 s as well as their activity level and their interactions with others. We also recorded whether they were using electronic media. To select subjects, the observer divided the playground into 3 zones based on dividing the total number of target areas in the playground by 3. Observers started each assessment by selecting a female child in the first zone and observing her for 8 min, divided into 24 20-s intervals. In each interval, the observer watched the visitor for 10 s and then took the next 10 s to record the location (target area), momentary physical activity level (i.e., sedentary, moderate, or vigorous) and interactions with others and with electronic media. This protocol was repeated in the second and third zones, with a female adult and then a male child, respectively, then going back to the first zone to observe a male adult and continuing to alternate the zone and the observed individual by age-group and gender. If the person of gender and age was not present in the preselected zone, the observer would then choose the next available gender or age-group or move to the next zone. The Play Loop protocol was conducted 3–4 h every day for 4 days at each playground.

We also documented the characteristics of each playground. We defined an atmosphere scale from 1 to 5 to describe the prevalence of plantings and trees: 1 was either no plantings or the presence of immature shrubs and low plantings inside the playground, 2 was mature shrubs inside the playground, 3 was immature trees inside the playground, 4 was mature trees directly outside the playground, and 5 was mature trees inside the playground. Other contextual characteristics we assessed included neighborhood poverty, playground size, and presence of restrooms.

To assess which features might be correlated with stay time in playgrounds, we identified the 10 most used by adult and child visitors in our national study, as indicated by the SOPARC protocol. Those features were swings, picnic tables, climbers, water play, slides, berms, towers, boulders, spinners, and platforms.

### 2.4. Data Analysis

We first calculated the descriptive statistics of the 60 study playgrounds, including both the playground and local neighborhood characteristics and the Dwell Time data. We used visitor groups as the analysis unit and reported descriptive statistics at the group level. We also calculated the descriptive statistics for electronic media use at the individual level. We used a repeated-measures multiple logistic regression model to assess the binary outcome of long vs. short stay and the contribution of explanatory variables, where playgrounds are clusters. The generalized estimating equation technique was applied to adjust for clustering. Sensitivity checks were conducted for alternative cutoffs including 15 and 30 min. Results were similar to the 35-min cutoff.

We fitted the statistical model to estimate the relationship between the duration of groups’ stay time and explanatory variables. Due to the high skewness of the duration, as well as the truncation at the maximum of 4 h of observation, we dichotomized the outcome variable to be relatively long versus short stay of a visitor group using a cutoff of 35 min, which is approximately at the sample mean, and between the median and the third quartile of the study sample. The explanatory variables included playground design and type, local neighborhood characteristics, playground features and design elements, and visitor group compositions. Both the outcome and all explanatory variables were objectively collected or retrieved from objective sources with no missing data.

Other control variables included the day of the week, time of day, and fixed effects for study cities, because prior studies suggested that park use can differ significantly between weekdays and weekends [20]. We implemented 2 sets of time variables for weekdays and weekends separately.

Given that the study was observational, it was deemed exempt from Human Subjects by the Institutional Review Board.

## 3. Results

### 3.1. Playground Characteristics

Among the 60 playgrounds, the mean population density of the neighborhood was 16,215 people within a half-mile radius. The average percentage of individuals in poverty was 13.7% (vs. 11.4% nationally) [21]. The average playground size was less than 1 acre (35,000 square feet) and had an average of 21.9 unique features and five different surface types. All the playgrounds had climbing features, with the average number per playground at 11.2 (range 2–31). Water play was a feature in 43% of playgrounds, and 80% had swings. Almost 62% had a nearby restroom, 63% had a drinking fountain, and 71.7% had an adjacent picnic area. Table 1 lists the prevalence and number of the 10 most used playground features across the 60 playgrounds based on the average number of users in playgrounds with these features, as measured in the SOPARC protocol.

### 3.2. Dwell Time Observations

Altogether, we observed 1456 groups of visitors entering and leaving the playground (total of 4278 people). Because the playgrounds had a wide variety of users who stayed for different periods of time, the average number of groups observed per playground was 24.3 (range 2–52). On average, the number of individuals per group was close to three (Table 2). Adults comprised 43.1% of those observed, with children comprising 48.3%. Among the children aged 12 and under, the largest proportion was 3–5 years old (21.7% of total people observed). Seniors over 60 were the smallest age-group represented (3.1%). There were relatively few teenagers (5.5%). There were more females (55.7%) than males (44.3%).

People stayed an average of 32 min in the playgrounds, but the range was wide, from 5 min to the entire 4 h of observed time. Stay time varied by group size, with larger groups staying longer.

### 3.3. Play Loop Observations and Use of Electronic Media

The sample of 3713 observed in the Play Loop protocol was similar to those users observed in the SOPARC protocol by gender and race/ethnicity, except more children were included than adults.

Overall, 40% of male adults and 45% of female adults were seen using electronic media during the 8-min Play Loop observations at least once, but on average used it during 37% and 34% of the observed intervals, respectively. In contrast, among children, only 6% of girls and 5% of boys used electronic media and did so on average for 30% and 22% of the observed intervals, respectively. Those never observed using electronic media engaged in 33–44% more moderate-to-vigorous activity (MVPA) among adults and 33–39% among children (Table 3).

### 3.4. Factors Associated with Duration of Stay Greater or Less than 35 Min

Table 4 shows factors associated with duration of stay in playgrounds. Features that increased the likelihood of staying more than 35 min included playground size: every additional 10,000 square feet increased the likelihood of staying longer by 20%. The presence of a restroom increased the likelihood of staying longer by 48%. Each increment on the five-unit atmosphere scale increased the likelihood of staying longer by 19%, so visitors to a playground with mature trees would be 94% more likely to stay more than 35 min. Every additional swing increased the likelihood of staying longer by 8% and every additional climber increased the likelihood of staying more than 35 min by 6%. Every additional spinner increased the likelihood of staying more than 35 min by 31%. The association between duration of stay and number of people in the group remained positive, even after controlling for all contextual factors.

Factors that reduced the likelihood of staying more than 35 min included the playground being located in a tourist destination, the number of platforms, and the number of picnic tables. If a teen was a part of the group observed, the group was 64% less likely to stay for 35 min or more. City, time, weekday and weekend control variables are not included in Table 4 for brevity. 

## 4. Discussion

This study identified playground features most associated with longer durations of playground visits, including the presence of restrooms, mature trees, larger playground size, swings, climbers, and spinners. Because playgrounds are outdoor settings and there are additional benefits for being outdoors, prioritizing the inclusion of these features in new playgrounds or in their renovations could make a difference in population-level health outcomes. There are logical reasons that these features support longer stays.

The presence of restrooms supported longer stays for families. Almost everyone needs to use a restroom during the day, and if there is not one on the premises, it is logical that visitors might leave sooner. The need for restrooms might be greater for children and older adults with overactive bladders [22].

Mature trees contribute to an atmosphere that provides shade and connections to nature, potentially leading to feelings of calmness, joy, and creativity and facilitating concentration [23,24]. Nature connectedness has been found to lower depression and anxiety [25].

Playground size may contribute to the length of stay because it supports more features, requires more time to move through the whole space, and increases physical activity due to longer distances traveled. The greater the variety of features that are visited may also increase the time needed to interact with each playground component. An extra half acre may increase the probability of staying longer than 35 min by at least 40%.

Swings, climbers, and spinners all support recommended muscle and bone strengthening activities [1] in addition to MVPA. The impact of optimally designed playgrounds may yield additional benefits for motor skill development [26], balance, and proprioception [27,28]. For instance, swings have been linked to better sleep [29]. Climbers can help people become more agile and flexible in their movements. Climbing involves stretching and building upper body grip and arm strength as well as core muscles. Spinners are features that increase MVPA, but also involve the vestibular and proprioceptive senses, increasing body awareness [28].

The high percentage of adults using electronic media and its association with lower levels of PA demonstrate its obvious impediment to physical activity, as attending to a screen interferes with being able to engage in other activities [30]. If the 8-min period in which people were observed represented the entire 32 min of an average visit duration, then playgrounds could contribute approximately 8.6 min of MVPA for adults using electronic media and 11.5–12.5 min for those not using it. Among children, playground visits could contribute an average of 15.4 min of MVPA for those using electronic media and 21.1 min for those not using it (Table 3). Being able to move and pay attention to surroundings is not possible while looking at electronic media at the same time.

### Limitations and Strengths

The limitations of the study have to do with the time that was used for observations, from 10 a.m. to 6 p.m., so we have no data gathered about evening use, a time that might be attractive to more teenagers or adults. Also, we only conducted observations during one week in the summer, which may not be representative of other weeks or other seasons. Further, we only visited the playgrounds on days when there was no precipitation.

The negative association with duration of stay and picnic tables is probably biased due to the timing of our observations. Dwell Time observations were scheduled at each park on Fridays and Saturdays from 10 a.m. to 2 p.m. and on Wednesdays and Sundays from 2 p.m. to 6 p.m. so that data collectors could have a lunch break at a reasonable time. Therefore, any group that came for lunch at noon, for example, would only be able to be observed until 2 p.m., which may have biased the overall findings. In a future study, the Dwell Time observations should have their start and end times more varied throughout the day.

Another limitation is the observation of movement within the playground being limited to 8 min. The lower levels of MVPA associated with the use of electronic media are derived from data that may not be fully representative of all the time individuals spend in the playground. It is possible that the same people might be more active once they completed their electronic media activity.

The sample of playgrounds was selected based on their being renovated or built in the last 10 years, and their locations are not fully representative of all US playgrounds. However, our goal was to understand more about different features that may be associated with physical activity, which necessitated choosing playgrounds that included a large variety of features.

Strengths of the study include a clustered design with playgrounds matched by neighborhood context and limiting the study to playgrounds of similar age and quality. We also had multiple observations over a week to capture daytime differences and multiple locations across the US. The use of direct observation is a well-validated methodology that is another strength of this study [18,31,32].

Future research directions include exploring expanded hours, as playgrounds may be used in the evenings, as well as expanding the seasons, to understand how time and weather influence MVPA, as well as the use of different playground features. In addition, including a wider variety of playgrounds may offer more insights into the importance of the quality of the features to document the influence of conditions on visit duration and MVPA. Exploring the use of electronic media further is also important to gauge whether its use dominates a playground visit or is only a small component. Given the average stay is over 30 min and our observation protocol related to electronic media was only 8 min, a longer protocol could reveal greater insights on electronic media use and its influence on MVPA. Finally, comparing the MVPA and physical and mental health among families by the frequency of playgrounds visits could provide important information on the benefits of promoting playground use.

## 5. Conclusions

Playground design is a factor that influences physical activity with respect to duration of time spent outdoors. Restrooms, the presence of mature trees, playground size, swings, climbers, and spinners stand out as the most important elements that support outdoor time and associated physical activity. Playground developers may also consider posting signs reminding visitors of the benefits of exercise, play, and being in nature, and advising people to take advantage of the playground structures, rather than using electronic media. Future renovations and playground designs should consider the most important features that support time spent outdoors and physical activity for all ages.

## Figures and Tables

**Table 1:** Descriptive characteristics of playgrounds.

	Playground and Neighborhood Characteristics Mean (Range)
Neighborhood Characteristics	
Neighborhood Population Density (sq f) (1/2 mile)	16,215.2 (410.7, 76,936.5)
% Individuals in Poverty (1/2 mile)	13.7 (2.8, 32.4)
Playground Characteristics	
% Destination Location, n (%)	14 (23.3%)
Avg. Playground Size (sq. ft.)	35,014 (4827.0, 108,089.0)
# Target Areas	70.7 (32.0, 151.0)
# Unique features	21.9 (14.0, 32.0)
Avg. presence of mature trees (atmosphere scale)	3.7 (0.0, 5.0)
Shade (sq feet)	16,430.2 (365.0, 74,821.0)
Sand (sq feet)	408.8 (0.0, 4127.0)
# with Restroom (%)	37 (61.7%)
# with Water Play features (%)	26 (43.3%)
# With Picnic Area (%)	43 (71.7%)
Avg # picnic tables/playground	2.9 (0.0, 20.0)
# with Climbing features (%)	60 (100%)
Avg # of climbing features/playground	11.2 (2.0, 31.0)
# Playgrounds with slides (%)	56 (93.3%)
Avg # slides/playground	3.4 (0.0, 9.0)
# with platforms (%)	53 (88.3%)
Avg # platforms/playground	4.5 (0.0, 14.0)
# with swings (%)	48 (80.0%)
Avg. Number of Swings/playground	5.2 (0.0, 21.0)
# with boulders (%)	32 (53.3%)
Avg # boulder features/playground	2.6 (0.0, 15.0)
# with spinners (%)	30 (50.0%)
Avg # spinners/playground	0.9 (0.0, 5.0)
# with berms (%)	27 (45.0%)
Avg # berms/playground	1.9 (0.0, 10.0)
# with towers (%)	10 (16.7%)
Avg # towers/playground	0.4 (0.0, 6.0)

N = 1456 Dwell Time groups excluding groups that had censored time (observer left before group), with demographic/time/target area data for N = 3684 group members, # Number.

**Table 2:** Descriptive characteristics of visitors and time spent in playground.

Variables	Average	Range
Number of groups observed per playground (N = 1456 groups)	24.3	2–52
Number of individuals observed (max 3 per group) per playground (N = 3684)	61.4	6–137
Average group size	2.94	1–18
Total number in groups	4278	-
Group Composition: Average # persons/group		
# infants (0–2) (%)	0.22 (7.6%)	0–2
# toddlers (3–5)	0.63 (21.7%)	0–4
# children (6–12)	0.55 (19.0%)	0–6
# teens (13–17)	0.16 (5.5%)	0–7
# adults (18–59)	1.25 (43.1%)	0–4
# seniors (60+)	0.09 (3.1%)	0–3
Group composition: Average # by gender		
Male	1.29 (44.3%)	0–7
Female	1.62 (55.7%)	0–7
Average visit duration (in minutes)	32.01	5–240
Innovatively designed playground	33.07	5–240
Traditional post-and-platform design	30.54	5–170
Average visit duration by gender (minutes)		
Male	31.48	5–240
Female	32.74	5–240
Average visit duration by group size (Minutes)		
Single (*n* = 67)	21.94	5–120
2 people (*n* = 550)	31.42	5–150
3 people (*n* = 498)	31.87	5–240
4 to 6 people (*n* = 315)	34.49	5–180
7 or more people (*n* = 26)	43.08	5–110

Of the 240 observation periods across the 60 playgrounds, there were only 8 periods when no groups were observed. Of the 232 observation periods where at least one group was observed, the mean number of groups was 6.3 (range 1–18, std 3.4), # Number.

**Table 3:** Percentage of electronic media users and time viewed using electronic media.

Viewed Using Electronic Media		MVPA	Sedentary	Time Using Electronic Media	Not Viewed Using Electronic Media	MVPA	Sedentary
Female Adult	N = 280 (45%)	121.9 (27%)	337.6	155.6 (34%)	N = 337	151.8 (36%)	274.5
Male Adult	N = 196 (40%)	123.8 (27%)	328.0	168.9 (37%)	N = 300	161.7 (39%)	256.3
Female Child	N = 74 (6%)	218.4 (49%)	223.2	97.0 (22%)	N = 1307	290.2 (65%)	156.3
Male Child	N = 58 (5%)	211.7 (48%)	231.0	133.1 (30%)	N = 1161	295.1 (67%)	146.2

**Table 4:** Model: factors associated with duration of playground visit.

Parameter	Estimate	Standard Error	Pr > |Z|
Intercept	−5.75	0.92	<0.0001
Innovative design	0.06	0.40	0.88
**Tourist destination**	**−0.67**	**0.31**	**0.03**
Population density/sq mile	−0.21	0.14	0.15
**Playground size (square feet)**	**0.02**	**0.01**	**0.03**
% families in poverty (0.5 square mile)	0.36	1.46	0.81
**Restroom present**	**0.48**	**0.20**	**0.02**
**Presence of mature trees (atmosphere scale)**	**0.19**	**0.08**	**0.02**
Shade (square feet)	−0.02	0.01	0.10
Sand (square feet)	0.18	0.12	0.14
Presence of water play features	0.13	0.26	0.63
**# swings**	**0.08**	**0.03**	**0.004**
**# platforms**	**−0.08**	**0.03**	**0.02**
# berms	0.01	0.06	0.86
# boulder features	−0.06	0.04	0.14
**# climbers**	**0.06**	**0.02**	**0.0008**
# slides	0.10	0.09	0.27
**# spinners**	**0.31**	**0.09**	**0.0004**
# towers	−0.05	0.09	0.56
**# picnic tables**	**−0.07**	**0.03**	**0.04**
**# persons per group**	**0.23**	**0.05**	**<0.0001**
Presence of children in group	0.31	0.24	0.20
**Presence of teen in group**	**−0.64**	**0.24**	**0.007**

Note: Significant findings are in bold, # Number.

## Data Availability

Data will be made publicly available within 6 months.
